# Robot Collection and Transport of Objects: A Biomimetic Process

**DOI:** 10.3389/frobt.2018.00048

**Published:** 2018-05-18

**Authors:** Daniel Strömbom, Andrew J. King

**Affiliations:** ^1^Department of Mathematics, Uppsala University, Uppsala, Sweden; ^2^Department of Biosciences, Swansea University, Swansea, United Kingdom

**Keywords:** bio-inspired robotics, feedback control, collective behavior, shepherding algorithm, adaptive system

## Abstract

Animals as diverse as ants and humans are faced with the tasks of collecting, transporting or herding objects. Sheepdogs do this daily when they collect, herd, and maneuver flocks of sheep. Here, we adapt a shepherding algorithm inspired by sheepdogs to collect and transport objects using a robot. Our approach produces an effective robot collection process that autonomously adapts to changing environmental conditions and is robust to noise from various sources. We suggest that this biomimetic process could be implemented into suitable robots to perform collection and transport tasks that might include – for example – cleaning up objects in the environment, keeping animals away from sensitive areas or collecting and herding animals to a specific location. Furthermore, the feedback controlled interactions between the robot and objects which we study can be used to interrogate and understand the local and global interactions of real animal groups, thus offering a novel methodology of value to researchers studying collective animal behavior.

## 1. Introduction

Predator attacks upon insect swarms, bird flocks, or fish schools provide a striking example of how one or a few agents (the predators) can influence the motion of many other agents (the prey) almost simultaneously (Hamilton, 1971; King et al., 2012; Handegard et al., 2012). Shepherding of sheep by dogs represents a caricature of this predator-prey interaction whereby the sheepdog maneuvers hundreds and sometimes thousands of livestock from one location to another (Strömbom et al., 2014). Engineers have long been fascinated by the act of shepherding and the behavioral rules that dogs adopt when herding since such knowledge may have application to engineering tasks as diverse as guiding groups of exploring robots (Turgut et al., 2008) to cleaning up the environment (Fingas, 2016). To this end, Strömbom et al. (2014) designed a general shepherding algorithm inspired by empirical data collected from real-life sheepdog interactions; it was proposed that the algorithm could support the efficient design of robots herding autonomous agents in a variety of contexts.

Research with multi-robot systems have sought to bring objects (and other robots) in the environment together as quickly as possible, into one cluster (Melhuish et al., 2001; Gauci et al., 2014), and such “herding” robot systems could have the potential to limit the spread of oil spills in the oceans (Zahugi et al., 2012; Fingas, 2016), and to collect rubbish (Bonnema, 2012), specific objects (Karunasena et al., 2008), or hazardous material (Nguyen et al., 2002) on both land and water. Whilst a large number of algorithms have been proposed for use in such tasks (Lien et al., 2004, 2005; Miki and Nakamura, 2006; Bennett and Trafankowski, 2012; Strömbom et al., 2014) most are studied via simulation and only capable of collecting or herding relatively low numbers of objects or agents, at least when only one shepherd is used (Bennett and Trafankowski, 2012). The use of robots for collection and herding objects in the real-world therefore remains rare, and herding free-living animals presents an even greater challenge, given that prey animals have evolved a variety of mechanisms to avoid detection and capture (Ioannou et al., 2012). In fact, the only published research we know to successfully apply a robot for herding free-living animals is work by Vaughan et al. (1998) who designed and used a robot to herd flocks of ducks.

Introducing robots into animal groups to influence/study the behavior of the animals has been much more common (and successful) in the field of collective animal behavior (Krause et al., 2011). Robots have been used to study the behavior of cockroaches (e.g. Halloy et al., 2007), fish (e.g. Faria et al., 2010; Swain et al., 2012; Landgraf et al., 2013, 2016; Cazenille et al., 2017) and rats (e.g. Shi et al., 2013). In most cases the interactions between the animals and the robot are essentially one-way; the animals are influenced by the robot but the robot is not directly influenced by the animals. However, examples do exist where two-way interactions between a robot and a group of animals are achieved. For example, in Swain et al. (2012) a feedback controlled robot-fish interacts with a school of free-moving fish in real time. The robot fish was programmed to chase the centroid of the fish school and dart towards them when their polarization was close to zero (milling or disordered school). Such examples demonstrate the potential for using robot-animal interactions, but to fully utilize robots in the study of collective behavior, the robots need to be able to respond to the real-life individuals (and not just the collective), in real-time (Krause et al., 2011).

To advance the study and analysis of robot-animal interactions requires an integrated design process (Hamann et al., 2016) that affords remotely controlled robots and 2d or 3d tracking of robot and object/animals. The task of fully automating the tracking of multiple objects can be “surprisingly problematic under experimental conditions” (Krause et al., 2011) but advances in image tracking technologies especially via open-source software (e.g. Pérez-Escudero et al., 2014) is making this more achievable. For example, the use of a surveillance drone providing a shepherding robot with information in real time about target objects or animals would revolutionize numerous cleanup processes, and enable robots to respond to their targets even when these targets are mobile or unpredictable in some way.

Here, we present an adaptive collection robot that is part of a feedback-controlled image-based tracking system designed to target and retrieve objects. The robot algorithm is an adapted version of Strömbom et al.’s (2014) bio-inspired model of shepherding behavior that matched empirical data collected with a sheepdog and sheep in the real-world when analyzed via computer simulations. We take the Strömbom et al. (2014) algorithm, modify it, and implement it in a single robot shepherd that collects and moves objects to a given location based on feedback from the image-based tracking. We demonstrate the collection capabilities of the robot in fixed and changing environments, and show that it is fully adaptive, robust to various sources of noise, and mimicks the sheepdog behavior on which it is based. We also explain why we believe that our algorithm is a viable candidate for implementation into suitable robots to collect and move living and artificial object in the real world and, crucially, how it can also be useful to study collective animal behavior via robots.

## 2. Material and  Methods

### 2.1. Test Arena

**Figure 1:**
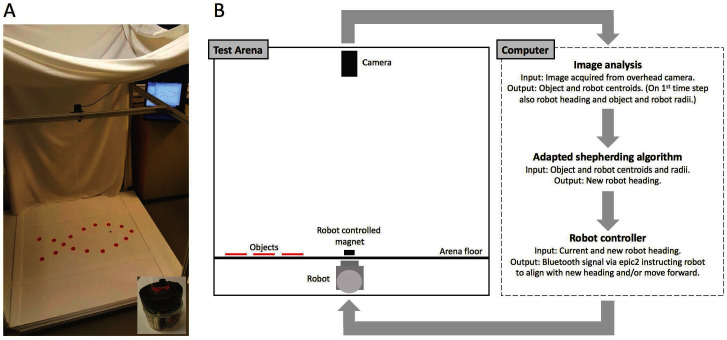
**(A)** Photo of the arena setup showing the white arena floor, red objects, black robot controlled magnet, overhead camera, and the computer used to coordinate and run all parts of the feedback control loop. Inset: e-puck robot fitted with a large red magnet used to connect to the small black magnet moving on the arena floor. **(B)** Schema illustrating the experimental setup.

We use an arena setup (Figure 1A,B) and feedback control loop similar to those employed in Swain et al. (2012) and Bonnet et al. (2017) to explore the capacity and behavior of an adapted shepherding algorithm implemented in an e-puck robot (Mondada et al., 2009) instructed to adaptively collect objects scattered in the arena to a designated collection zone. The arena floor is made out of acrylic and boundaries of the same material have been set up limiting the space available for herding to 880 × 435 mm. In our set-up, the robot moves under the arena floor and controls the movement of a black magnet (radius 5 mm) which interacts with red round objects (radius 15 mm) via physical contact on the arena floor. The robot is connected to the computer via Bluetooth and instructions are transmitted to it via the e-puck Matlab control application ePic2 (Hubert and Weibel, 2008).

### 2.2. Feedback Control Loop

We use an overhead camera (Logitech C902 HD pro USB) linked to a computer running Matlab R2015b. The camera takes an image, which is processed and the coordinates and radii of the objects and the robot controlled magnet are extracted using elementary image processing and analysis. The current (time t) normalized orientation/heading of the robot ${\hat{H}}_{t}$, radius of the objects ($r_{o}$), and the radius of the robot magnet ($r_{r}$) are also calculated.  The centroid coordinates and the radii of the objects and robot magnet are then used to calculate a new robot heading ${\hat{H}}_{t+1}$ for the next time step using the shepherding algorithm which is described in 2.2.2 below. The process continues until all N objects have been delivered to the collection zone which is a discshaped region near the center of the arena with radius $4+0.5N^{2/3}r_{o}$ (Figure 1B).

#### 2.2.1. Image Processing and Analysis

We chose to use red objects, a black robot controlled magnet, and a white arena floor because this enabled fast, low-level image processing analysis methods on low resolution images (640 × 480). Here we describe the steps involved in the image analysis, and when applicable, include the Matlab command used in parenthesis following the description. Once an image has been imported to Matlab we overexpose it slightly and then segment the black and red objects by simple thresholding. A morphological operation is then applied to fill any “holes” in the segmented objects (*imfill*) and the centroids of the segmented objects are then calculated (*regionprops centroid*). Finally, the areas of the robot magnet and an object in the image are estimated by counting object pixels in the segmented images (*nnz*) and from these areas the radius of the robot magnet $r_{r}$ and the radius $r_{o}$ of the objects are calculated. As the objects do not change size the radii are only calculated on the first time step of each trial. At the beginning of each trial the current heading (in arena coordinates) ${\hat{H}}_{0}$ of the robot is estimated by extracting the centroids of the robot magnet in two successive webcam photos, acquired while the robot is moving straight ahead in its local coordinate system.

#### 2.2.2. The Shepherding Algorithm

**Figure 2:**
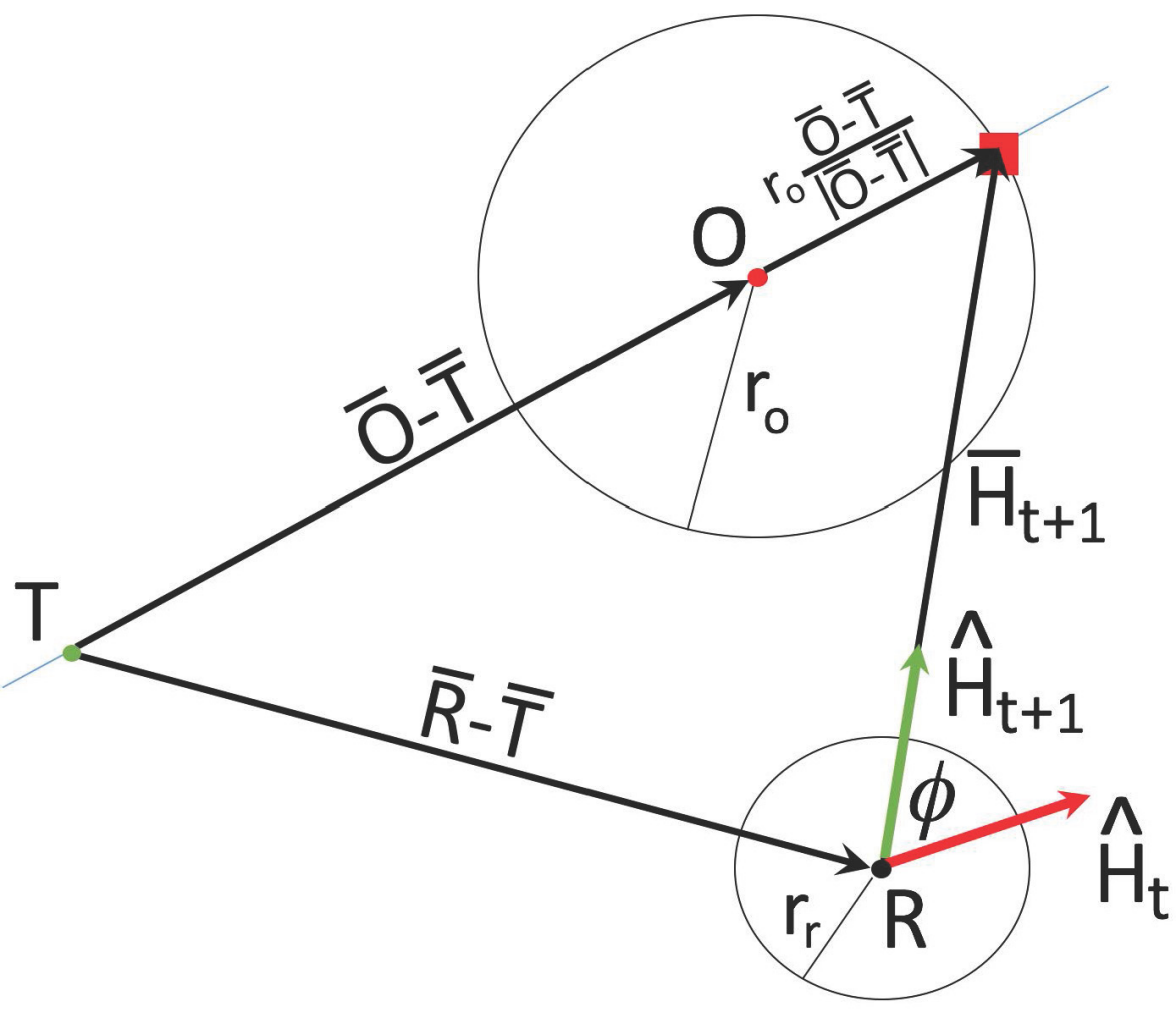
Geometry of the collection algorithm. The green dot represents the center of the collection zone and is denoted by T. The red dot represents the centroid of the object to be collected, and we denote it by O, and the circle surrounding it at a distance of $r_{o}$ represents the object boundary. The black dot represents the centroid of the robot, denoted by R, and the circle at a distance of $r_{r}$ from it represents the robot magnet boundary. The red vector ${\hat{H}}_{t}$ is the current heading of the robot, the green vector is the new heading ${\hat{H}}_{t+1}$ the robot should move in to approach the collection point (red square) on the far side of the object relative to the target, and ϕ is the angle between the current and new heading.

The shepherding algorithm is modified from the collection part of the algorithm in Strömbom et al. (2014), adapting it for use with non-self-propelled objects with contact repulsion. The algorithm is designed to collect the object furthest away from the collection zone first, unless it is already in contact with another object, in which case it delivers that object to the collection zone first before venturing out towards the furthest away object. Figure 2 illustrates how the new robot heading ${\hat{H}}_{t+1}$ is calculated from known quantities once a specific object has been selected for collection. We use hat notation for unit vectors and bar notation for non-normalized vectors. T denotes the center of the collection zone, O the centroid of the object to be collected, and R the centroid of the robot. The new heading of the robot is set towards the point on the object boundary on the far side of the centroid of the object O relative to the target T. This point is represented by a red square in Figure 2 and we see that the new heading vector from the robot towards this point is given by

(1) $${\bar{H}}_{t+1}=\left(\bar{O}-\bar{T}\right)+r_{o}\frac{\bar{O}-\bar{T}}{|\bar{O}-\bar{T}|}-\left(\bar{R}-\bar{T}\right)=\bar{O}-\bar{R}+r_{o}\frac{\bar{O}-\bar{T}}{|\bar{O}-\bar{T}|}.$$

Once the algorithm has calculated a new heading ${\bar{H}}_{t+1}$ for the robot, the signed angle ϕ between the normalized current heading ${\hat{H}}_{t}$ and the normalized new heading ${\hat{H}}_{t+1}$ is calculated (Figure 2). If the magnitude of this angle is smaller than a specified threshold (0.25 radians ≈ 14 degrees), the robot controller instructs the robot to keep moving forward, otherwise the controller rotates the robot into alignment with the new heading before moving forward. Once the robot has been moved the loop starts over and a new photo is taken by the overhead webcam. This process continues until all objects present on the arena floor has been delivered to a pre-assigned collection zone.

#### 2.2.3. Experiments

We conducted a series of experiments to investigate the collection capacity and behavior of the robot. We examined situations with a fixed number of objects to be collected (phase one) and situations where the number of objects changed over time (phase two). In phase one, we ran four trials each with 2, 4, 8 and 16 objects. Objects were distributed in the arena so that no object was in the collection zone or touching an arena boundary initially. In phase two, three trials were conducted, and in each case the number of objects for collection increased within the trial. Phase two trials started with two objects, and then we added two more, then four more, and finally eight more. Objects were added to the arena once the robot was driving the final object in the arena (i.e., the 2nd, 4th, and 8th object) towards the collection zone. Trials where objects tossed into the arena ended up in the collection zone were excluded. Across all trials (both phases) the robot always started near the center of the arena and each trial terminated when all objects had been delivered to the collection zone. We collected the coordinates of the robot and the objects throughout the trials and the time to completion of each trial was recorded.

#### 2.2.4. Measures

To evaluate the collection capacity of the robot and characterize the collection process we constructed time series with (i) the mean object-target distances, and (ii) the area occupied (convex hull) by objects. To evaluate the behavioral mechanisms by which the robot herded and collected objects we also recorded (iii) where the robot was located relative to the position of the object being herded and final target destination. To this end, we expressed the coordinates of the robot centroid in a coordinate system that is centered on the centroid of the closest object and in which the direction towards the target is the positive x-axis. More specifically, on each time step we determine if the robot is within a distance of $2r_{o}$ (our definition of close) from any object and if so proceed with steps 1–3 below.

1. First specify that the centroid of the object O is the origin of the new coordinate system and then translate the robot centroid R and target coordinate T accordingly. That is, $R^{\prime }=R-O$ and $T^{\prime }=T-O$.
2. Calculate the (signed) angle θ of the origin to target vector.
3. Rotate the translated robot vector $R^{\prime }$ and the translated target vector $T^{\prime }$ by θ.

## 3. Results

### 3.1. Robot Performance in Task

**Figure 3:**
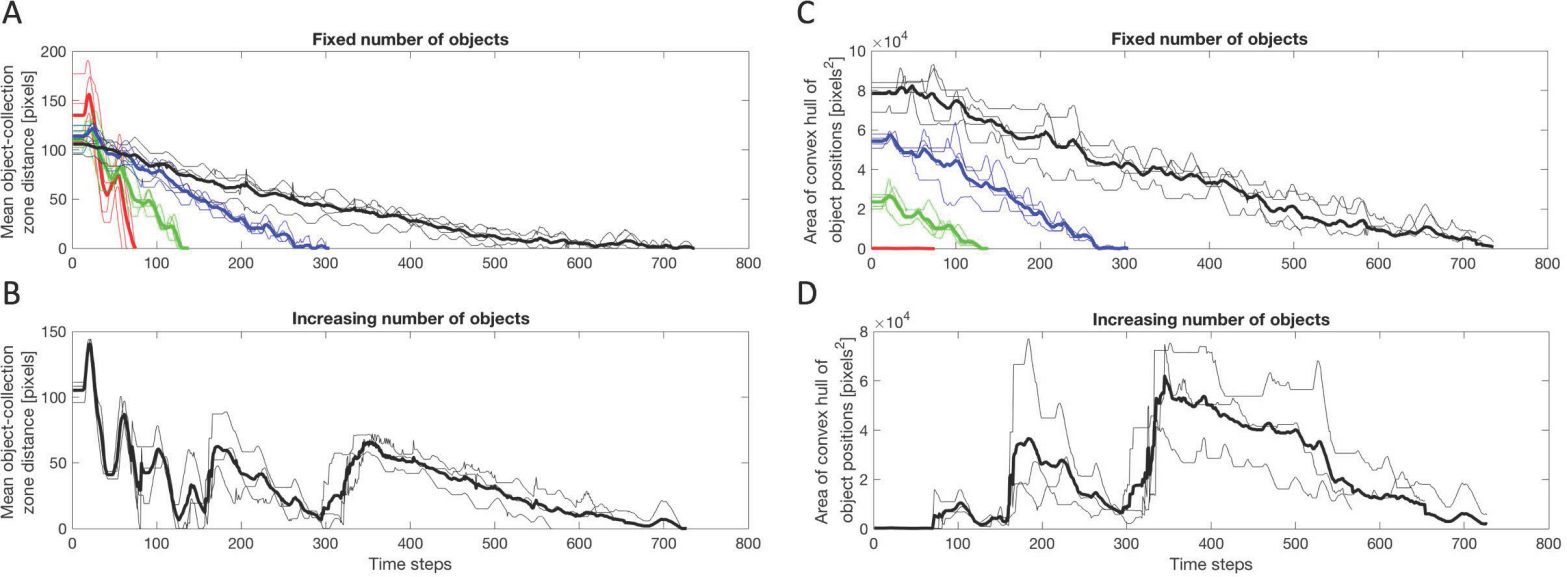
**(A–B)** Mean object-collection zone distance over time. Thin lines show the mean distance through time in each individual trial and thick lines the mean over all trials with that numberof objects. **(A)** With fixed number of 2 (red), 4 (green), 8 (blue) and 16 (black) objects. **(B)** With increasing number of objects. **(C–D)** Area of convex hull of object positions over time (for N=2 distance is used). Thin lines show the area of the convex hull through time in each individual trial and thick lines the mean over all trials with that number of objects. When calculating the mean over all trials the area of the convex hull of a trial that has finished is set to 0. **(C)** With fixed number of 2 (red), 4 (green), 8 (blue) and 16 (black) objects. **(D)** With increasing number of objects.

Examples of the robot collection process are provided in Video S1 which shows one collection trial each for 2, 4, 8 and 16 objects, and one trial with an increasing number of objects. All objects and zones shown in Video S1 have been  superimposed on the webcam image: Target (blue asterisk), Collection zone (green ring), Object centroids (red asterisks), Object boundaries (red ring), Robot controlled magnet centroid (black asterisk), Robot controlled magnet boundary (black ring), Current heading (red rod), New (ideal) heading (green rod). The mean average distance of objects to the collection zone (Figure 3), and the dispersion of the objects as described by a convex hull (Figure 3C,D) during trials illustrate the performance of the robot for fixed and variable number of object trials.

Figure 3A We found the completion times across trials for a fixed number of objects were similar (Figure 3A) and mean completion and standard deviation (time steps) for 2 objects = 68.3±7.3, 4 objects = 130.0±5.6, 8 objects =273.8±20.2, and 16 objects = 670.5±108.0.  Figure 3A,C also confirms that the initial configurations of objects were different in each trial as the initial average object to collection zone distances and convex hulls are different. The relatively low variation in completion times and the fact that initial configurations were different suggests that the process is robust with respect to the initial configurations of objects.

By comparing Figure 3A,B (and Figure 3C,D) we see that the mean completion time for the case of fixed N=16 and the case with an increasing number of objects are similar. In addition, by comparing the time evolution of the process we see that the process with an increasing number of objects reaches the milestones 2, 4 and 8 objects around the same time that the corresponding fixed number of object trials finishes. This suggests that the process is adaptive with respect to changes in the number of objects, and that potential time and/or efficiency losses associated with its operation in the case of an increasing number of objects versus a fixed number of objects are small.

### 3.2. Robot Behavior

**Figure 4:**
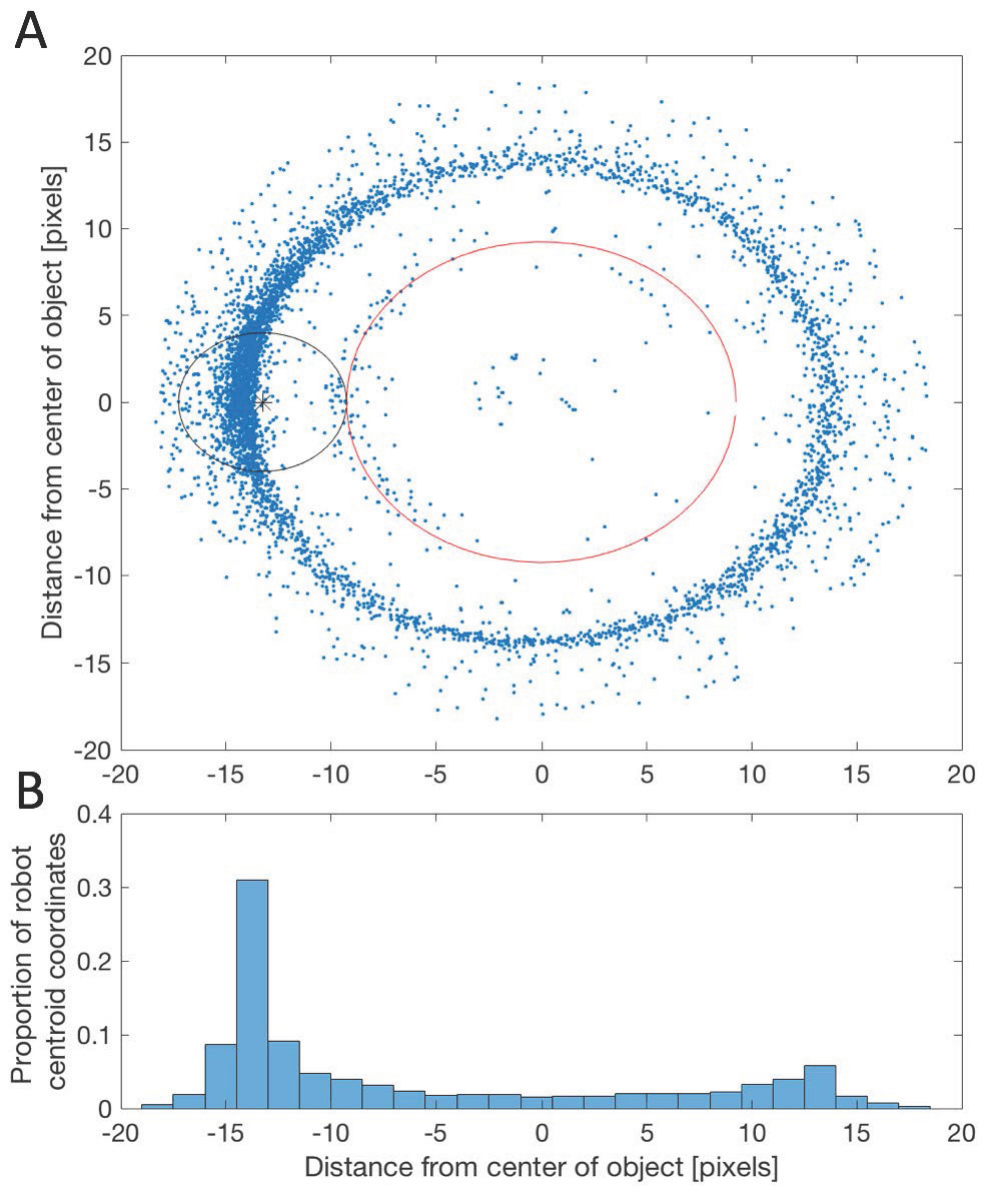
**(A)** Position of robot when near an object relative to the direction of the target (here the positive x-axis). On top of the scatter plot of robot centroid coordinates (blue points) we have inserted a larger red circle representing the object ($r_{o}=9.25$ pixels) and a smaller black circle representing the robot magnet ($r_{r}=4$ pixels).** (B)** Relative frequency histogram of robot x-coordinates when near an object.

Robot-object interactions are dominated by appropriate collection maneuvers by the robot. When close to an object (within $2r_{o}$) the robot spends a majority of the time directly behind it relative to the target as presented in Figure 4A where the a majority of the robot centroids (blue dots) are on the far side of the object relative to the target. In particular, there is a dense cluster of robot centroids with x-coordinates ranging from about −13 to −15 (Figure 4A), which  appears to be the ideal position from which to drive the object to the target (Figure 4B). Indeed, the peak at +13 to +15 in Figure 4B shows that when the robot is on the same side of the object as the target it often pushes the object directly away from the target while attempting to get around it. This is also reflected in the short increases before linear decreases in the measures provided in Figure 4A,B. This phenomenon is a consequence of the fact that when the robot is initially approaching an object it often comes directly from the collection zone having just delivered another object. Note that there are some blue dots closer to the object than the object and robot radii should allow, and in some cases even apparently inside the object. These are the result of rare occasions when the robot magnet partially of fully slip up on top of the object. These situations typically sort themselves out quickly and the robot magnet gets off and continues to push the object within a few time steps. However, if the process is supervised inducing a small perturbation to the object or robot can help resolve it even faster.

## 4. Discussion

We have shown that our biomimetic collection algorithm works when implemented into a simple robot and that the resulting robot collection process exhibits several potentially useful properties.

The collection process is robust with respect to the initial configurations of objects, in the sense that differences in initial configuration of objects does not lead to large differences in completion time (Figure 3A,C). This result therefore indicates that this process may be a good candidate for reliable collection of objects in novel and noisy environments. In addition, the process is adaptive with respect to changes in the number of objects (Figure 3A,C). So it may operate in a changing environment as well as fixed. Finally, there are no obvious time and/or efficiency losses associated with its operation in a changing environment as compared to a fixed environment (comparing Figure 3A,B, and Figure 3C,D) which would suggest that the cost of operation in a changing environment is effectively the same as in a fixed environment.

We have established that the robot-object interactions are dominated by appropriate collection maneuvers by the robot (Figure 4), and that the resulting robot behavior is consistent with the behavior exhibited by sheepdogs and simulated shepherds herding sheep/agents (cf. Figure 5ab, Strömbom et al., 2014). In particular, comparing Figure 5ab from Strömbom et al., 2014 with Figure 4B presented in our results, shows that the real dog (Figure 5a, Strömbom et al., 2014), the simulated shepherd (Figure 5b, Strömbom et al., 2014), and the robot (Figure 4B) all exhibit distance from the center of flock/object distributions that are skewed with one dominant peak. That the robot-object interactions are dominated by appropriate collection maneuvers by the robot shows that the underlying algorithm and the implementation into the robot are robust with respect to noise. We know that there are several sources of noise/error in our experimental trials, which are, in order of estimated impact: (i) the robot controlled magnet is not fixed exactly at the center of the robot but has some flexibility, (ii) fluctuations in the time it takes to send instructions to the robot from Matlab via Bluetooth, (iii) image acquisition (any variation in lighting conditions) and centroid calculation error, and (iv) noise in the electrical components (in particular the robot itself). Moreover, whilst our robot does not “behave optimally” (e.g., the robot sometimes pushes gathered objects outside of the collection zone when on route to collect others) its operation is robust and it does, on average, perform well. For our purposes, this is a positive result because it reflects a reality of biological systems, and we did not set out to minimise a cost function (Pérez-Escudero et al., 2009).

Due to the above listed properties of the collection process and its implementation into this simple e-puck robot, in particular its robustness and adaptability, we believe that the algorithm presented here could potentially be used to reliably and effectively collect objects from the environment both on land and on the surface of water if implemented into an appropriate robot. To directly use the implementation presented here, including the feedback control loop, the robot could work as part of a pair, with a surveillance drone that provides the collection robot with overhead images. Considering how accessible advanced drone technology is today this should not present an obstacle. Such a pair consisting of one collection/guiding robot and one surveillance drone could potentially solve a number of problems that are impossible, dangerous, and/or costly for humans to deal with directly. For example, moving animals from sensitive areas (DeVault et al., 2011), removing or limiting the spread of oil on water (Zahugi et al., 2012; Fingas, 2016), collecting hazardous materials (Nguyen et al., 2002), guiding people to safety in areas/rooms with low visibility (Isobe et al., 2004), and potentially even for evacuation and rescue from disaster sites (Patterson et al., 2013).

Finally, we expect that integrating our approach of emphasizing two-way robot-individual interactions into advanced frameworks for animal-robot interactions (e.g. Swain et al. 2012; Bonnet et al. 2017), will afford a greater integration of function and mechanism in the study of collective animal behavior. In particular, it would allow the use of robots to investigate phenomena thought to be intimately linked with specific identifiable individuals, e.g., influential leaders (Jiang et al., 2017). For example, using a robot with two-way interaction would allow for a precise and dynamic manipulation of leadership traits (played out by a robot) enabling a more standardized, repeatable experimental design and causal analysis of leader-follower dynamics (Nakayama et al., 2012) and their consequences for group-level patterns of behaviour (Cazenille et al., 2017).

## Author Contributions

AK and DS planned and designed the study, and wrote the paper. DS constructed the arena and feedback control loop, performed the experiments, and processed and analyzed the data.

## Conflict of Interest Statement

The authors declare that the research was conducted in the absence of any commercial or financial relationships that could be construed as a potential conflict of interest.

## Supplementary material

Video S1 Shows one collection trial each for 2, 4, 8 and 16 objects and one trial with an increasing number of objects. All relevant calculated quantities have been superimposed on the overexposed webcam image used as input to the image analysis part. These quantities are: Target (blue asterisk), Collection zone (green ring), Object centroids (red asterisks), Object boundaries (red ring), Robot controlled magnet centroid (black asterisk), Robot controlled magnet boundary (black ring), Current heading (red rod), New (ideal) heading (green rod).
